# Availability of anticancer medicines in public and private sectors, and their affordability by low, middle and high-income class patients in Pakistan

**DOI:** 10.1186/s12885-017-3980-3

**Published:** 2018-01-03

**Authors:** Muhammad Rehan Sarwar, Sadia Iftikhar, Anum Saqib

**Affiliations:** 1 Department of Pharmacy, The Islamia University of Bahawalpur, Bahawalpur, Punjab Pakistan; 2 Akhtar Saeed College of Pharmaceutical Sciences, Lahore, Pakistan

**Keywords:** Cancer, Anticancer medicines, Availability, Affordability, Originator brand, Lowest price generics

## Abstract

**Background:**

Availability and affordability of anticancer medicines is a matter of great concern especially for low and middle income countries e.g., Pakistan. Prime focus of this study was to evaluate the availability of anticancer medicines in public and private sectors, and their affordability among patients with different income levels.

**Methods:**

A descriptive, cross-sectional survey was conducted in 22 cancer care hospitals (18 public hospitals and 04 private hospitals) and 44 private pharmacies in Punjab, Pakistan. All (*n* = 4400) participants were ≥18 years of age. Data were collected at different intervals and analyzed by using Statistical Packages for Social Sciences (IBM SPSS Statistics for Windows, Version 21.0. Armonk, NY: IBM Corp.)

**Results:**

A total of 4913 patients were approached, and 4400 responded to the survey (response rate = 89.6%). Non-hodgkin lymphoma (12.3%), breast cancer (8.6%), and leukemia (7.6%) were the most prevailing cancers. Conventional medicines like cisplatin, cyclophosphamide, and etoposide were the most prescribed medicines. Oncologists were reluctant to prescribe newer anticancer medicines due to high prices. Originator brands (OBs) were more readily available (52.5%) but less affordable (53.4%); whereas, lowest price generics (LPGs) were less available (28.1%) but more affordable (67.9%). Anticancer medicines were more affordable by the high income class patients than the low income class patients.

**Conclusion:**

The availability of both OBs and LPGs was greater at private hospitals and pharmacies as compared to public hospitals. The high income class had more affordability of both OBs and LPGs; however, LPGs were more affordable for all income classes.

## Background

Cancer is amongst the most expensive and lethal non-communicable diseases globally [1]. In 2016, the most prevailing cancers in Pakistan were breast cancer (21.8%), leukemia (6.3%), hodgkin lymphomas (4.9%) and non-hodgkin lymphoma (4.7%) of the total reported cases [2]. However, the actual prevalence of cancer may be greater than this due to lack of availability of proper registry system in Pakistan. Presently, the management of cancer mainly relies upon the availability and affordability of anticancer medicines. In recent years, the emergence of newer anticancer medicines has rapidly and substantially caused an expansion not only in the repertoire but also in the average per month cost of these therapeutic agents. Cancer treatment demands substantial cost i.e., ranging from $4500 to >$10,000 per month [3, 4], thus posing huge burden on patient and healthcare system.

The heath sector of Pakistan is regulated by the provincial governments. The government health coverage is inadequate and negligible in terms of public health insurance and employer benefits. Therefore, majority of the population have to bear their health expenses on their own [5]. In 2004, a “National Action Plan for Prevention and Control of Non-Communicable Diseases and Health Promotion” [6] was developed with the collaboration of World Health Organization (WHO). This plan was designed to cover various aspects e.g., capacity enhancement of healthcare system, up-gradation of cancer registration, and making an organizational network at local, provincial and national levels. For accomplishing all such goals the WHO cancer coordinator for Pakistan has also developed a National Cancer Control Council. Because of financial constraints the government of Pakistan was unable to contribute in this program and all strategies were merely dependent on the funding from the WHO [7].

Several factors which affect the accessibility of any therapeutic agent have an impact on patient’s pocket and subsequently cause a considerable delay in the commencement of therapy [8]. Some of these factors may include (a) the extent to which a drug is reimbursed or subsidized, (b) the allocation of budget by the public sector for the purchase of medicines, (c) licensing of medicines for manufacturing and import, (d) implication of evidence-based guidelines, and (e) procurement by the government hospitals and insurers [9]. The need of pre-approval for the provision of subsidized medicines and “mark-up values” by the hospitals, wholesale dealers, pharmacists, and physicians may also contribute in making the prices extremely high [10, 11].

Pricing of medicines in Pakistan are regulated by the Drug Regulatory Authority of Pakistan (DRAP) which works under Federal government, though no transparent price calculation formula is mentioned in the Drugs Act, 1976 [12]. According to a survey conducted by the WHO, the prices of originator brands (OBs) and lowest-price generics (LPGs) were 3.36 and 2.26 times more than the international retail price in Pakistan. Moreover, a sudden rise in price of 15% in November 2013 further burdened the patients [13].

The affordability of anticancer medicines is a grave problem for most of the Pakistani patients. Since 45.5% of the Pakistani population lives below the poverty line [14] so the expenses pertaining to healthcare are unaffordable for an average income person. The availability and affordability of anticancer medicines in Pakistan are surrounded by evidence based three common issues which include: (i) formulary limitations; anticancer medicines have not been mapped in the form of formulary, (ii) actual availability; inadequate provision of health services due to shortage or poor availability of medicines [13, 15] and (iii) the barriers like resources and affordability associated with the access of newer anticancer medicines. Moreover, inflation (Consumer Price Index (CPI) inflation: 1.3% on year-on-year basis in September 2015) and low affordability leads to an underuse of effective medicines. Despite of several measures adopted by the Ministry of National Health Services, regulations and coordination of affordability of medicines is still a problem owing to the expansion of OBs, and ongoing variation and inconsistency of prices of medicines in the country. The availability of essential generic medicines is only 15% and 31% in the public and private sector healthcare facilities, respectively. Even though the LPGs are used but still the cost of treatment for chronic illnesses is unaffordable for middle-income and low-income people of Pakistan [16–18]. This holds true not only for Pakistan but for other countries as well. A study conducted across 49 European countries elucidated that there are disparities in the availability of cancerous medicines, which are responsible for their inequitable access [19].

The unavailability or unaffordability issues would not only aggravate the underlying disease but also lead to the inequities between the patients. Up till now, numerous studies focusing on the gravity of underlying problems have been conducted in multiple countries, excluding Pakistan. The aim of current study is to assess the availability of anticancer medicines in public and private sectors, and their affordability by high, middle, and low-income class patients.

## Methods

### Study design and settings

A descriptive, cross-sectional study design was employed. There are total 23 (18 public and four private sector tertiary care) hospitals in Punjab province of Pakistan which provide services to cancer patients. Out of these 23 hospitals, seven were specialized cancer-care hospitals. One hospital was excluded from the survey because it provides services solely to the pediatrics. Survey was carried out in 22 cancer-care hospitals and 44 private pharmacies in Punjab, a province of Pakistan. Data were collected from the pharmacies and cancer patients attending selected hospitals and evaluated according to the objectives of study.

### Study population and sample size

The population under study was cancer patients aged ≥18 years, who visited the selected cancer-care hospitals for routine examinations. According to the latest Pakistani census, the population of the surveyed province consisted of 101,391,000 individuals [20]. The minimum sample size was 4147 as calculated by the Raosoft sample size calculator [21] based on cancer prevalence in Pakistan. With contingency of 5% for non-response and inappropriate responses, the final sample was calculated to be 4400.

### Data collection and outcome variables

A total of 4913 cancer patients were approached over a six month period (1st January, 2017 to 30th June, 2017), 4400 patients consented to participate (response rate = 89.6%). Data was collected at different intervals from the selected cancer-care hospitals.

A data collection form was designed for this study which consisted of three main parts: (1) socio-demographic characteristics, (2) diagnosis and (3) recommended medicines. The reliability of the survey tool was assessed by conducting a pilot study. Piloting was undertaken using data from 100 patients. After piloting, the data collection form was restructured.

### Measurements

#### Socio-demographic characteristics

Socio-demographic characteristics given in Table 1 were recorded for each participant. Those participants who were retired (taking pension) or running a business were classified as employed and housewives were considered as unemployed. The data was obtained through face to face questioning of patients. To avoid biasness, the data regarding employment status and income level of the participants was validated by using online tax payer verification system of Federal Board of Revenue (FBR) [22].

#### Diagnosis and prescribing pattern

The type of cancer and all the medicines present in each prescription were noted on a pre-designed performa sheet. Anticancer medicines having more than one active ingredient were not evaluated. The most commonly prescribed anticancer medicines were categorized according to the prescribing trend; low (prescribed to <5% of the selected patients), medium (prescribed to ≥5% of the selected patients but <10%) and high (prescribed to >10% of the selected patients).

#### Availability of anticancer medicines and their per month cost

Forty anticancer medicines were chosen for the survey. These anticancer medicines were selected on the basis of, (a) pilot study in which local needs and cancer burden was assessed, (b) literature review, and (c) the opinions of various experts. During the survey, if medicines were present at the pharmacy settings then they considered as available.

The availability of anticancer medicines was evaluated in public hospitals, private hospitals, and private pharmacies. For the assessment of prices associated with these medicines, *Pharmaguide 2016,* was consulted [23]. The process of data collection was done by trained pharmacy students under the supervision of survey manager and principal investigator. Principal investigator checked the collected and completed Performa’s on weekly basis. If any information was found missing then a follow up visit to the respective setting was conducted. Before initiation of the process of data collection, medical superintendents/directors were contacted by the principal investigator. In this way a good cooperation was established between the team of investigators and the staff members of the selected settings. To avoid report biasness (e.g. up coding, less availability of medicine to gain attention for budget increase, etc.), the drugs were said to be available if they were present in the settings and the patients could avail them on prescription. Also, the formulary list and purchase records were assessed for data validation. For each medicine, data were collected on the basis of per unit price, and availability of OBs and LPGs. On the basis of standard guidelines and the recommended treatment, per unit price of anticancer agents were transformed into per month cost.

Furthermore, the following criteria were used to describe the availability of medicines:

*Absent:* 0% of facilities: these medicines were not found in any facility surveyed;

*Low*: <50% of facilities: these medicines were hard to find;

*Fairly high*: 50–74% of facilities: these medicines were available in many facilities;

*High*: >75% of facilities: good availability.

### Affordability of anticancer medicines

According to the WHO and Health Action International (HAI) methodology, for the assessment of affordability we have to calculate that “the income of how many days is required to purchase the medicines for 30 days (in case of chronic condition e.g. cancer)”. Generally, if the total cost of therapy for 1 month is equal to or less than the wage of 1 day then it is said to be affordable.

A study published by Rasha Khatib et al. [24] defined it as; “if the combined cost of therapy is <20% of household capacity-to-pay then it can be considered as affordable.” In this study this concept modified and affordability was measured for each prescribed medicine by low, middle, and high income class of patients through this formula;

$$ Affordability=\frac{\%\ast \mathrm{of}\  \mathrm{household}\  \mathrm{capacity}\  \mathrm{to}\ \mathrm{pay}}{Per\  month cost of the medicine}\times 100 $$

* If 1 medicine was prescribed it was 20%, if 2 medicines were prescribed it was 10%, if 3 medicines were prescribed it was 6.7% and if 4 medicines were prescribed it was 5% of household capacity to pay.

### Statistical analysis

Statistical Package for Social Sciences (IBM, SPSS Statistics for Windows, version 21.0. Armonk, NY: IBM Corp.) was used for data analysis. Descriptive statistics such as frequencies, percentages, and mean were used to present the data.

## Results

Four thousand four hundred cancer patients were investigated in the study. Just over half (55.4%, *n* = 2436) of the participants were male, and 39.3% (*n* = 1731) were aged 18–39 years. 67.9% (*n* = 2987) were married, 67.8% (*n* = 2981) had secondary education level and 40.7% (*n* = 1791) had income status of upper class. 61.9% (*n* = 2723) respondents were employed and three-quarters (73.2%, *n* = 3291) were urban residents (Table 1).

**Table 1:** Characteristics of the study population

Variables		Male (*n* = 2436)	Female (*n* = 1964)	Total (*n* = 4400)
		n (%)	n (%)	n (%)
Age (years)	18–39	959 (39.4)	772 (39.3)	1731 (39.3)
	40–64	780 (32.0)	866 (44.1)	1646 (37.4)
	≥65	697 (28.6)	326 (16.6)	1023 (23.3)
Civil Status	Single	84 (3.4)	43 (2.2)	127 (2.9)
	Married	1722 (70.7)	1265 (64.4)	2987 (67.9)
	Widowed	370 (15.2)	514 (26.2)	884 (20.1)
	Divorced	260 (10.7)	142 (7.2)	402 (9.1)
Education level	Primary (≤10 years)	503 (20.6)	0 (0.0)	503 (11.4)
	Secondary (11–13 years)	1389 (57.0)	1592 (81.1)	2981 (67.8)
	Tertiary (≥14 years)	544 (22.3)	372 (18.9)	916 (20.8)
Annual income	Low class (PKR0–299,999)	662 (27.2)	481 (24.5)	1143 (26.0)
	Middle class (PKR300,000–999,999)	842 (34.6)	624 (31.8)	1466 (33.3)
	Upper class (PKR ≥ 1,000,000)	932 (38.3)	859 (43.7)	1791 (40.7)
Employment Status	Employed	2138 (87.8)	585 (29.8)	2723 (61.9)
	Unemployed	298 (12.2)	1379 (70.2)	1677 (38.1)
Residence	Rural	855 (35.1)	326 (16.6)	1181 (26.8)
	Urban	1581 (64.9)	1638 (83.4)	3219 (73.2)
Number of medicines	1	67 (2.8)	157 (8)	224 (5.1)
	2	1768 (72.6)	1204 (61.3)	2972 (67.5)
	3	559 (22.9)	532 (27.1)	1091 (24.8)
	4	42 (1.7)	71 (3.6)	113 (2.6)

The most common cancers diagnosed among participants were; non-hodgkin lymphoma (NHL) (12.3%, *n* = 540), breast cancer (8.6%, *n* = 378) and leukemia (7.6%, *n* = 334) (Table 2).

**Table 2:** Cancer cases diagnosed in the study population

Sr. No	Cancer	*ICD-10*	Male (*n* = 2436)	Female (*n* = 1964)	Total (*n* = 4400)
			n (%)	n (%)	n (%)
1	Bladder	*C67*	42 (1.7)	32 (1.6)	74 (1.7)
2	Brain	*C70–72*	111 (4.6)	70 (3.6)	181 (4.1)
3	Breast	*C50*	–	378 (19.2)	378 (8.6)
4	Cervix uteri	*C53*	–	142 (7.2)	142 (3.2)
5	Colorectal	*C18–21*	131 (5.4)	43 (2.2)	174 (4.0)
6	Corpus uteri	*C54*	–	71 (3.6)	71 (1.6)
7	Gallbladder	*C23–24*	110 (4.5)	71 (3.6)	181 (4.1)
8	Hodgkin lymphoma	*C81*	149 (6.1)	43 (2.2)	192 (4.4)
9	Kidney	*C64–66*	178 (7.3)	114 (5.8)	292 (6.6)
10	Larynx	*C32*	111 (4.6)	70 (3.6)	181 (4.1)
11	Leukemia	*C91–95*	221 (9.1)	113 (5.8)	334 (7.6)
12	Lip, oral cavity	*C00–08*	110 (4.5)	70 (3.6)	180 (4.1)
13	Liver	*C22*	111 (4.6)	70 (3.6)	181 (4.1)
14	Lung	*C33–34*	186 (7.6)	104 (5.3)	290 (6.6)
15	Non-Hodgkin lymphoma	*C82–85*	363 (14.9)	177 (9.0)	540 (12.3)
16	Esophagus	*C15*	107 (4.4)	71 (3.6)	178 (4.0)
17	Ovary	*C56*	–	141 (7.2)	141 (3.2)
18	Pancreas	*C25*	111 (4.6)	70 (3.6)	181 (4.1)
19	Prostate	*C61*	220 (9.0)	–	220 (5.0)
20	Stomach	*C16*	65 (2.7)	43 (2.2)	108 (2.5)
21	Thyroid	*C73*	110 (4.5)	71 (3.6)	181 (4.1)

The most commonly prescribed anticancer medicines were: cisplatin (49.5%, *n* = 2177), etoposide (25.8%, *n* = 1137), and cyclophosphamide (19.9%, *n* = 877). The detailed description about the prescribed anticancer medicines is given in Table 3.

**Table 3:** Anticancer medicines prescribed to study participants

Sr. No	Medicine and Dose	ATC Code	f (*n* = 9893) %^a^	Trend	OB	Per month cost	LPG	Per month cost
1	Anastrozole 1 mg tab	L02BG03	71 (1.6)	Low	Anastrozole *(Novartis)*	6000	Femizet *(Atco)*	5130
2	Bicalutamide 50 mg tab	L02BB03	109 (2.5)	Low	Casodex *(ICI)*	12,642	Calutide *(A. J. Mirza)*	4308
3	Bleomycin 15 mg inj	L01 DC01	42 (1.0)	Low	Bleomycin *(Pharmedic)*	9960	Bemocin *(Atco)*	8000
4	Capecitabine 500 mg tab	L01 BC06	258 (5.9)	Medium	Xeloda *(Roche)*	25,000	NA	NA
5	Carboplatin 150 mg inj	L01XA02	206 (4.7)	Low	Carpsol *(Pfizer)*	6681	Carboplatin *(Atco)*	3000
6	Cisplatin 50 mg inj	L01XA01	2177 (49.5)	High	Cisplasol *(Pfizer)*	3099	Platosin *(Pharmachemie)*	1750
7	Cyclophosphamide 500 mg inj	L01AA01	877 (19.9)	High	Cyclomide *(Pharmedic)*	5625	Cyclophosphamide *(S. Ejazuddin)*	3000
8	Cyproterone Acetate 50 mg tab	G03HA01	66 (1.5)	Low	Androcur *(Bayer)*	3588	NA	NA
9	Cytarabine 100 mg inj	L01 BC01	342 (7.8)	Medium	Cytosar *(Pfizer)*	2700	Cytarabine *(Highnoon)*	1785
10	Dacarbazine 200 mg inj	L01AX04	74 (1.7)	Low	Duticin *(Al-Habib)*	2700	Darbazine *(Pharmedic)*	2500
11	Dactinomycin 0.5 mg inj	L01DA01	71 (1.6)	Low	Dactinomycin (*Al-Habib)*	28,616	Dactinofin *(Pharmedic)*	23,520
12	Daunomycin 20 mg inj	L01DB02	111 (2.5)	Low	Daunoblastina *(Pfizer)*	6750	D-Blastin *(Pharmedic)*	5700
13	Docetaxil 80 mg inj	L01CD02	18 (0.4)	Low	Taxotere (*Sanofi aventis)*	76,000	Docekebir *(Oncogene)*	74,400
14	Doxorubicin 50 mg inj	L01DB01	385 (8.8)	Medium	Adriblastina *(Pfizer)*	4495	Doxorubicin *(Al- Habib)*	3170
15	Epirubicin 50 mg inj	L01DB03	427 (9.7)	Medium	Farmorubicin *(Pfizer)*	13,270	Anthracin *(Atco)*	9510
16	Etoposide 100 mg inj	L01CB01	1137 (25.8)	High	Etoposide *(Pfizer)*	7150	Lymphoside *(CCL)*	4950
17	Fludarabine phosphate 50 mg inj	L01BB05	180 (4.1)	Low	Fludara *(Sanofi aventis)*	66,313	Fludakebir *(Oncogene)*	46,400
18	Flourouracil 500 mg inj	L01 BC02	502 (11.4)	High	Pharmauracil *(Pharmedic)*	1944	Secouracil *(S. Ejazuddin)*	176
19	Gemicitabine 1 g inj	L01 BC05	685 (15.6)	High	Gemzar *(Eli lilly)*	64,020	Gemita *(Atco)*	41,650
20	Hydroxyurea 500 mg cap	L01XX05	43 (1.0)	Low	Hydra *(Medinet)*	1200	Hydrine *(Al-Habib)*	1168
21	Ifosfamide I gminj	L01AA06	71 (1.6)	Low	Ifosfamin *(Pharmedic)*	12,000	Fosfamin *(CCL)*	12,000
22	Imatinibmesylate 400 mg tab	L01XE01	67 (1.5)	Low	Glivec *(Novartis)*	140,000	NA	NA
23	Irinotecan 100 mg inj	L01XX19	181 (4.1)	Low	Campto *(Pfizer)*	133,480	Irinocan *(Pharmedic)*	71,250
24	Lapatinib 250 mg tab	L01XE07	43 (1.0)	Low	Tykerb *(GSK)*	201,650	NA	NA
25	Letrozole 2.5 mg tab	L02BG04	71 (1.6)	Low	Femara (*Novartis)*	8720	Letara (*A.J. Mirza)*	5100
26	Mercaptopurine 50 mg tab	L01BB02	111 (2.5)	Low	Mercaprine *(Pharmedia)*	741	Purinetone *(Al- Habib)*	630
27	Methotrexate 10 mg tab	L01BA01	72 (1.6)	Low	Emthexate *(Pharmachemie)*	817	Unitrexate *(Al-Habib)*	410
28	Mitomycin 10 mg inj	L01 DC03	71 (1.6)	Low	Mitocin *(Pharmedic)*	2256	Mitomycin *(S.Ejazuddin)*	232
29	Mitoxantrone 20 mg inj	L01DB07	43 (1.0)	Low	Mitoxantrona (Atco)	4000	NA	NA
30	Nilotinib 200 mg cap	L01XE08	43 (1.0)	Low	Tasigna *(Novartis)*	456,000	NA	NA
31	Oxalplatin 100 mg inf	L01XA03	288 (6.5)	Medium	Oxitan *(Atco)*	60,000	Eloxatin *(Sanofi aventis)*	52,500
32	Paclitaxel 260 mg inf	L01CD01	71 (1.6)	Low	Intaxel *(Atco)*	34,600	Paclixil *(A.J. Mirza)*	34,600
33	Pazopanib 400 mg tab	L01XE11	67 (1.5)	Low	Votrient *(GSK)*	176,666	NA	NA
34	Sorafenib 200 mg tab	L01XE05	43 (1.0)	Low	Nexavar *(Bayer)*	465,600	NA	NA
35	Sunitinib 50 mg cap	L01XE04	67 (1.5)	Low	Sutent *(Pfizer)*	392,640	NA	NA
36	Tamoxifen 20 mg tab	L02BA01	71 (1.6)	Low	Tamox *(Pharmedic)*	600	Tamooxe *(Al-Habib)*	450
37	Thalidomide 100 mg cap	L04AX02	43 (1.0)	Low	Thalido*(Atco)*	6000	NA	NA
38	Vinblastine 10 mg inj	L01CA01	42 (1.0)	Low	Velbastine *(Al-Habib)*	4165	Vinblas *(Pharmedic)*	2800
39	Vincristine 2 mg inj	L01CA02	522 (11.9)	High	Pharmacristine *(Pharmedic)*	1580	Vincristine Gador *(Seignior)*	1124
40	Vinorelbine 50 mg inj	L01CA04	71 (1.6)	Low	Vinelbine *(Atco)*	33,480	Vinkebir *(Oncogene)*	33,480

^a^Percentages given with respect to the total sample size of patients. ATC = Anatomical Therapeutic Chemical; f = Frequency; OB = Originator brand; LPG = Lowest price generic; NA = Not available. *Note:* The specialists were reluctant to prescribe medicines such as bevacizumab, cabazitaxel, cetuximab, erlotinib, idarubicin, pemetrexed, rituximab, ruxolitinib, temozolomide, topotecan, and trastuzumab due to their much higher prices

### Availability of anticancer medicines (originator brands and lowest price generics)

The mean availability of anticancer medicines in both public and private sectors was found to be 52.5% for OBs, while 28.1% for LPGs. Furthermore, study revealed a fairly high availability for OBs while generally low availability for LPGs. The availability of Fluorouracil (97%), Etoposide (95.5%), Methotrexate (95.5%) and Tamoxifen (95.5%) was maximal among the OBs; whereas, Gemicitabine (81.1%), Bleomycin (56.1%) and Doxorubicin (56.1%) had the highest availability amongst LPGs in all study settings (see Table 4).

**Table 4:** Availability of anticancer medicines in public and private sectors in Punjab, Pakistan

Sr. No	Medicine and Dose	Public hospitals (*n* = 18)		Private hospitals (n = 4)		Private pharmacies (*n* = 44)		All (*n* = 66)	
		OB	LPG	OB	LPG	OB	LPG	OB	LPG
1	Anastrozole 1 mg tab	0 (0.0)	0 (0.0)	1 (25.0)	0 (0.0)	4 (9.1)	2 (4.5)	5 (7.6)	2 (3.0)
2	Bicalutamide 50 mg tab	0 (0.0)	0 (0.0)	1 (25.0)	0 (0.0)	3 (6.8)	1 (2.3)	4 (6.1)	1 (1.5)
3	Bleomycin 15 mg inj	10 (55.6)	8 (44.4)	4(100)	2 (50.0)	41 (93.2)	27 (61.4)	55 (83.3)	37 (56.1)
4	Capecitabine 500 mg tab	4 (22.2)	NA	4 (100)	NA	21 (47.7)	NA	29 (44.0)	NA
5	Carboplatin 150 mg inj	13 (72.2)	5 (27.8)	4(100)	1 (25.0)	33 (75.0)	29 (65.9)	50 (76.0)	35 (53.0)
6	Cisplatin 50 mg inj	8 (44.4)	9 (50.0)	3 (75.0)	0 (0.0)	43 (97.7)	11 (25.0)	54 (82.0)	20 (30.3)
7	Cyclophosphamide 500 mg inj	15 (83.3)	3 (16.7)	4 (100)	0 (0.0)	41 (93.2)	9 (20.5)	60 (91.0)	12 (18.2)
8	Cyproterone Acetate 50 mg tab	7 (38.9)	NA	4 (100)	NA	39 (88.6)	NA	50 (76.0)	NA
9	Cytarabine 100 mg inj	3 (16.7)	0 (0.0)	3 (75.0)	1 (25.0)	34 (77.3)	7 (15.9)	40 (61.0)	8 (12.1)
10	Dacarbazine 200 mg inj	4 (22.2)	1 (5.6)	2 (50.0)	1 (25.0)	27 (61.4)	13 (29.5)	33 (50.0)	15 (22.7)
11	Dactinomycin 0.5 mg inj	8 (44.4)	2 (11.1)	3 (75.0)	1 (25.0)	42(95.5)	31 (70.5)	53 (80.3)	34 (51.5)
12	Daunomycin 20 mg inj	2 (11.1)	0 (0.0)	1 (25.0)	1 (25.0)	16(36.4)	11 (25.0)	19 (29.0)	12 (18.2)
13	Docetaxil 80 mg inj	7 (38.9)	0 (0.0)	3 (75.0)	0 (0.0)	33(75.0)	7 (15.9)	43 (65.1)	7 (10.6)
14	Doxorubicin 50 mg inj	14 (77.8)	4 (22.2)	4 (100)	2 (50.0)	44(100)	31(70.5)	62 (94.0)	37 (56.1)
15	Epirubicin 50 mg inj	2 (11.1)	3 (16.7)	4 (100)	1 (25.0)	14(31.8)	9 (20.5)	20 (30.3)	13 (19.7)
16	Etoposide 100 mg inj	15 (83.3)	0 (0.0)	4 (100)	0 (0.0)	44(100)	13 (29.5)	63 (95.4)	13 (19.7)
17	Fludarabine phosphate 50 mg inj	0 (0.0)	0 (0.0)	4 (100)	1 (25.0)	11(25.0)	4 (9.9)	15 (23.0)	5 (7.6)
18	Flourouracil 500 mg inj	16 (88.9)	0 (0.0)	4 (100)	2 (50.0)	44(100)	19 (43.2)	64 (97.0)	21 (31.8)
19	Gemicitabine 1 g inj	5 (27.8)	11 (61.1)	4 (100)	2 (50.0)	31(70.5)	41 (93.2)	40 (61.0)	54 (81.8)
20	Hydroxyurea 500 mg cap	7 (38.9)	0 (0.0)	3 (75.0)	0 (0.0)	23(52.3)	11 (25.0)	33 (50.0)	11 (16.7)
21	Ifosfamide I gminj	5 (27.8)	2 (11.1)	2 (50.0)	0 (0.0)	19(42.3)	21 (47.7)	26 (39.4)	21 (31.8)
22	Imatinibmesylate 400 mg tab	0 (0.0)	NA	3 (75.0)	NA	15(34.1)	NA	18 (27.3)	NA
23	Irinotecan 100 mg inj	0 (0.0)	0 (0.0)	2 (50.0)	0 (0.0)	11(25.0)	10 (22.7)	13 (20.0)	10 (15.2)
24	Lapatinib 250 mg tab	0 (0.0)	NA	2 (50.0)	NA	13(29.6)	NA	15 (23.0)	NA
25	Letrozole 2.5 mg tab	0 (0.0)	0 (0.0)	2 (50.0)	0 (0.0)	16(36.4)	8 (18.2)	18 (27.2)	8 (12.1)
26	Mercaptopurine 50 mg tab	13 (72.2)	3 (16.7)	4 (100)	0 (0.0)	41(93.2)	11 (25.0)	58 (88.0)	14 (21.2)
27	Methotrexate 10 mg tab	15 (83.3)	1 (5.6)	4 (100)	1 (25.0)	44(100)	19 (43.2)	63 (95.4)	21 (31.8)
28	Mitomycin 10 mg inj	2 (11.1)	0 (0.0)	2 (50.0)	1 (25.0)	31(70.5)	12 (27.3)	35 (53.0)	13 (19.7)
29	Mitoxantrone 20 mg inj	0 (0.0)	NA	3 (75.0)	NA	18 (40.9)	NA	21 (32.0)	NA
30	Nilotinib 200 mg cap	0 (0.0)	NA	3 (75.0)	NA	19 (43.2)	NA	22 (33.3)	NA
31	Oxaliplatin 100 mg inf	1 (5.6)	0 (0.0)	2 (50.0)	2 (50.0)	21 (47.7)	11 (25.0)	24 (36.4)	13 (19.7)
32	Paclitaxel 260 mg inf	11 (61.1)	4 (22.2)	3 (75.0)	1 (25.0)	44 (100)	23 (52.3)	58 (88.0)	28 (42.4)
33	Pazopanib 400 mg tab	0 (0.0)	NA	1 (25.0)	NA	4 (9.1)	NA	5 (7.6)	NA
34	Sorfenib 200 mg tab	0 (0.0)	NA	1 (25.0)	NA	5(11.4)	NA	6 (9.1)	NA
35	Sunitinib 50 mg cap	0 (0.0)	NA	0 (0.0)	NA	3 (6.8)	NA	3 (4.5)	NA
36	Tamoxifen 20 mg tab	15 (83.3)	2 (11.1)	4 (100)	2 (50.0)	44(100)	16 (36.4)	63 (95.4)	20 (30.3)
37	Thalidomide 100 mg cap	4 (22.2)	NA	4 (100)	NA	25 (56.8)	NA	33 (50.0)	NA
38	Vinblastine 10 mg inj	9 (50.0)	3 (16.7)	4 (100)	0 (0.0)	31(70.5)	19 (43.2)	44 (67.0)	34 (51.5)
39	Vincristine 2 mg inj	11 (61.1)	2 (11.1)	3 (75.0)	0 (0.0)	33 (75.0)	23 (52.3)	47 (71.2)	25 (37.9)
40	Vinorelbine 50 mg inj	0 (0.0)	0 (0.0)	2 (50.0)	2 (50.0)	21 (47.7)	11 (25.0)	23 (35.0)	13 (19.7)
Total		31.4%	11.7%	71.9%	20.0%	59.4%	34.9%	52.5%	28.1%

OB = Originator brand; LPG = Lowest price generic; NA = Not available

### Affordability at different income levels

The affordability of anticancer medicines (OBs and LPGs) by high, middle, and low-income class patients is listed in Table 5. Patients with high income level could afford the expenditures on anticancer medicines; reverse was true for low income level patients. The most affordable LPGs (afforded by 100% patients) for low income class patients include Cytarabine, Flourouracil, Mercaptopurine, Methotrexate, Mitomycin and Tamoxifen, respectively.

**Table 5:** Affordability of anticancer medicines by high, middle and low-income class patients in Punjab, Pakistan

Sr. No.	Medicine and Dose	OB			Overall OB	LPG			Overall LPG	Overall both (OB + LPG)
		High	Middle	Low		High	Middle	Low		
1	Anastrozole 1 mg tab	100	50.1	18.3	71.5	100	68.3	20.6	63.2	68.2
2	Bicalutamide 50 mg tab	100	28.2	12.4	48.9	100	100	73.8	93.3	66.4
3	Bleomycin 15 mg inj	68.3	18.1	6.5	27.8	78.8	46.6	15.4	46.9	36.0
4	Capecitabine 500 mg tab	61.9	25.0	8.1	35.5	NA	NA	NA	NA	35.5
5	Carboplatin 150 mg inj	100	93.1	32.7	79.3	100	100	77.1	93.6	85.0
6	Cisplatin 50 mg inj	100	100	60	91.6	100	100	97.5	99.3	94.4
7	Cyclophosphamide 500 mg inj	100	65.5	28.0	70.5	100	95.9	57	86.6	77.0
8	Cyproterone Acetate 50 mg tab	100	99.5	43.8	84.5	NA	NA	NA	NA	84.5
9	Cytarabine 100 mg inj	100	100	69.9	92.5	100	100	100	100	94.7
10	Dacarbazine 200 mg inj	100	77.8	53.6	78.3	100	100	67.9	90	83.4
11	Dactinomycin 0.5 mg inj	100	42.6	14.7	66.1	100	51.8	17.8	56.4	62.3
12	Daunomycin 20 mg inj	100	50.3	15.7	58.1	100	100	43.3	85.6	69.0
13	Docetaxil 80 mg inj	31.8	5.5	3.2	15.1	NP	NP	NP	NP	15.1
14	Doxorubicin 50 mg inj	100	62.5	27.8	68.7	100	93.5	39.5	80.3	73.0
15	Epirubicin 50 mg inj	75.3	28.6	9.0	44.2	96.4	38.1	19.1	55.3	48.6
16	Etoposide 100 mg inj	100	77.4	31.0	74.6	100	92.4	43.9	82.1	77.6
17	Fludarabine phosphate 50 mg inj	30.1	8.2	2.8	15.9	40.3	11.1	5.1	2.7	17.8
18	Flourouracil 500 mg inj	100	100	60.9	90.6	100	100	100	100	94.4
19	Gemicitabine 1 g inj	21.3	9.7	3.5	13.2	32.4	13.5	5.2	18.1	15.1
20	Hydroxyurea 500 mg cap	100	100	100	100	NP	NP	NP	NP	100
21	Ifosfamide I gminj	62.9	29.5	9.1	42.3	62.9	29.2	8.8	33.5	38.8
22	Imatinibmesylate 400 mg tab	7.2	2.4	0.8	3.6	NA	NA	NA	NA	3.6
23	Irinotecan 100 mg inj	6.7	2.2	1.0	3.8	11.6	4.4	2.1	6.5	4.9
24	Lapatinib 250 mg tab	8.0	3.0	1.0	5.1	NA	NA	NA	NA	5.1
25	Letrozole 2.5 mg tab	86.5	40.7	12.6	58.3	100	68.7	20.7	63.3	60.3
26	Mercaptopurine 50 mg tab	100	100	100	100	100	100	100	100	100
27	Methotrexate 10 mg tab	100	100	100	100	100	100	100	100	100
28	Mitomycin 10 mg inj	100	100	48.5	89.2	100	100	100	100	93.5
29	Mitoxantrone 20 mg inj	100	100	52.5	90.1	NA	NA	NA	NA	90.1
30	Nilotinib 200 mg cap	2.2	0.9	0.3	1.4	NA	NA	NA	NA	1.4
31	Oxalplatin 100 mg inf	14.5	5.8	2.0	8.2	18.0	6.6	3.5	10.3	9.1
32	Paclitaxel 260 mg inf	23.9	8.5	4.6	15.6	23.9	14.6	5.0	14.5	15.1
33	Pazopanib 400 mg tab	14.8	3.5	1.4	6.8	NA	NA	NA	NA	6.8
34	Sorafenib 200 mg tab	6.3	3.2	1.1	4.3	NA	NA	NA	NA	4.3
35	Sunitinib 50 mg cap	6.7	1.6	0.6	3.1	NA	NA	NA	NA	3.1
36	Tamoxifen 20 mg tab	100	100	100	100	100	100	100	100	100
37	Thalidomide 100 mg cap	100	67.7	24.3	75.1	NA	NA	NA	NA	75.1
38	Vinblastine 10 mg inj	100	43.2	15.6	50.5	100	100	43.9	81.3	63.7
39	Vincristine 2 mg inj	100	100	85.2	96.3	100	100	99.4	99.8	97.6
40	Vinorelbine 50 mg inj	100	8.7	4.8	54.6	100	15.1	5.1	39.2	48.5
Total		70.7	49.1	29.2	53.4	84.4	69.6	49.0	67.9	55.5

OB = Originator brand; LPG = Lowest price generic; NA = Not available; NP: Not prescribed

## Discussion

The initial step for cancer control and prevention is to develop the proper understanding of relationship between disease and demographics [25]. This study reported 73.2% of the cases from urban areas and 26.8% from rural areas. Many cancer cases remained undiagnosed in Pakistan due to financial obstacles and poor availability of health care facilities (e.g. inadequate system of population based registers, and deprived diagnosis as well as treatment facilities in rural areas as compared to urban areas) [26, 27]. Therefore, exact number of cancer cases might be far greater in number than that of reported.

Breast cancer (19.2%) was the most commonly diagnosed cancer among females while NHL (14.9%) was commonly found in males. Such a high prevalence of breast cancer is not only found in Pakistan, it can be seen throughout the world. It is estimated that nearly half of all the reported cases of breast cancer and 38% of all the deaths due to this fatal illness have been reported from developed countries. The various subtypes of NHL are thought to alter immune system and show different pattern of incidence.

### Availability of anticancer medicines (originator brands and lowest price generics)

The availability of anti-cancerous medicines is mandatory for saving lives of cancer patients. In many low and middle income countries (LMICs) the availability of LPGs is often less [28] e.g., a cross-sectional study conducted in Dar es Salaam (Tanzania) revealed that the availability of anticancer drugs in healthcare settings was 50% of the total surveyed medicines while only 30% of the patients could get the anticancer drugs from the healthcare settings [29]. Similarly, the current findings showed that in both sectors the overall availability of OBs (52.5%) can be considered as fairly high in comparison with the LPGs (28.1%). Most of the OBs are the products of the multinational pharmaceutical companies (MPCs). These MPCs adopt various strategies (e.g., promotional techniques and the patent rights) in order to compete with the local pharmaceutical companies (LPCs). Due to the limitation of resources, LPCs cannot manage budget for promotional strategies. The promotional efforts of MPCs make product well-familiar to the prescribers. Therefore, prescribers are compelled to prescribe these medicines. According to the Trade-Related Aspects of Intellectual Property Rights (TRIPS) agreement, Pakistan has a right to include in its patent legislation a provision to manufacture LPGs without the requisition of any consent from the patent holder since Pakistan is a member of World Trade Organization (WTO). But, it was also found that 10 medicines LPGs were not available in the market.

In LMICs, the availability of medicines in the healthcare settings is considerably influenced by the cost [30]. This study revealed that the availability of these anticancer medicines was high in the private sector (71.9% for OBs and 20.0% for LPGs) as compared to the government healthcare settings (31.4% for OBs and 11.7% LPGs). Due to financial constraints, the government of Pakistan is unable to maintain good infrastructure of the public healthcare settings [31]. Thus government hospitals often face the issue of unavailability or shortage of medicines as compared to private sectors.

Unlike the conventional medicines new anticancer medicines were less readily available in both sectors. In LMICs like Pakistan, the retail prices are the major deterrent to access when compared with the cost at the supplier level [32]. In Pakistan, the high taxation associated with these lifesaving medicines is a cruel joke with the cancer sufferers. All the national and international organizations i.e., the WHO, HAI, The United States Agency for International Development (USAID), United Nations Organization (UNO) and DRAP must provide adequate funding so that tax free anticancer medicines can be made available to the local masses.

### Affordability of anticancer medicines at different income levels

In Pakistan, the affordability of medicines, especially anticancer medicines, is widely affected by the proliferation of OBs [12]. Our findings showed that the LPGs (67.9%) are more affordable than the OBs (53.4%). Because of price constraints medicines are not 100% affordable for general public, so OBs were found to be more affordable (70.7%) for high income patients, less affordable (49.1%) for middle income patients, and least affordable (29.2%) for low income patients. This may cause a great risk of disease progression, higher rate of mortalities and morbidities. In this study, the overall affordability for both OBs and LPGs was found to be 55.5% which makes cancer a catastrophic disease for local masses [33]. Another dilemma of LMICs is that the local masses are unaware of the importance of health insurance [34]. But sometimes these insurance policies fail to provide benefits or demand substantial co-payment [35]. Private health insurance schemes cover medicines cost. But high inflation, low per capta income and increasing cost of living are among the several hurdles that hinder the individuals for buying private health insurance and pay monthly premium. The government hospitals of Pakistan do not require any copayment for consultation and medicines. But in private hospitals all the expenses have to be paid by the patient [36]. Therefore, in 2014 Pakistani government took initiative in the form of Prime Minister National Health Insurance Program. This program aimed to cover a large number of cancer sufferers in both government and private sector. But without the cooperation of international organizations, this program cannot cover all the financially constrained civilians of Pakistan.

### Strength and limitations

There is no previously published study that evaluates the anticancer medicines with respect to availability in public and private sectors, and affordability with respect to income class especially in LMICs like Pakistan. Our study will provide a door to the researchers of other LMICs to evaluate availability and affordability related barriers towards optimal cancer treatment in their respective settings so that cancer medicines can be made affordable all over the entire globe.

There are some limitations in this study. First, the availability was measured at ‘one time’ on the day of data collection from any health facility. Therefore some facilities might usually have a product is available, but the drug may be out of stock on the day of data collection. Second, although this paper contains data on availability of anticancer drugs in Pakistan but it does not give insight in to what extent current guidelines of drug treatment of cancer are compromised by limited access to anticancer drugs. So, we cannot conclude what the effect of this is to outcome of anticancer treatment in Pakistan patients. Third, the authors measured households’ capacity to pay by collecting household income information, though it is often recommended that household ordinary expenditure excluding durable goods consumption will better reflect household’s capacity to pay.

## Conclusion

Cancers like non-hodgkin lymphomas and breast cancer are prevalent in Pakistan. The study revealed a fairly high availability for OBs and generally low availability for LPGs. The availability of these agents is greater in private sector as compared to public sector. The overall affordability of LPGs is more as compared to OBs irrespective of the income class; however, both of them are more affordable by high income class patients. Government and regulatory authorities must take adequate steps and formulate such policies to ensure the equitable availability and affordability of cancer medicines to fight against this deadly disease.

## Abbreviations

HAI: Health Action International
LPCs: Local Pharmaceutical Companies
LPGs: Lowest Price Generics
MPCs: Multinational Pharmaceutical Companies
OBs: Originator Brands
SPSS: Statistical Packages for Social Sciences
WHO: World Health Organization
